# High‐dose vitamin D metabolite delivery inhibits breast cancer metastasis

**DOI:** 10.1002/btm2.10263

**Published:** 2021-10-27

**Authors:** Jiaye Liu, Junyi Shen, Chunyang Mu, Yang Liu, Dongsheng He, Han Luo, Wenshuang Wu, Xun Zheng, Yi Liu, Sunrui Chen, Qiuwei Pan, Yiguo Hu, Yinyun Ni, Yang Wang, Yong Liu, Zhihui Li

**Affiliations:** ^1^ Department of Thyroid and Parathyroid Surgery West China Hospital, Sichuan University Chengdu China; ^2^ Laboratory of Thyroid and Parathyroid diseases, Frontiers Science Center for Disease‐Related Molecular Network, West China Hospital Sichuan University Chengdu China; ^3^ State Key Laboratory of Biotherapy and Cancer Center, West China Hospital Sichuan University and Collaborative Innovation Center Chengdu China; ^4^ Respiratory Health Institute Frontiers Science Center for Disease Molecular Network, West China Hospital, Sichuan University Chengdu China; ^5^ Department of Liver Surgery & Liver Transplantation Center West China Hospital, Sichuan University Chengdu China; ^6^ Department of Pharmaceutics, School of Pharmacy China Pharmaceutical University Nanjing China; ^7^ Department of Rheumatology and Immunology, Rare Disease Center, West China Hospital Sichuan University Chengdu China; ^8^ Shanghai OneTar Biomedicine Shanghai China; ^9^ Department of Gastroenterology and Hepatology Erasmus MC‐University Medical Center Rotterdam The Netherlands; ^10^ Department of Medical Biochemistry and Biophysics Karolinska Institute Stockholm Sweden; ^11^ Department of Gastroenterological Surgery West China Hospital, Sichuan University Chengdu China

**Keywords:** breast cancer, calcitriol, drug delivery, metastasis, vitamin D

## Abstract

Besides its well‐known benefits on human health, calcitriol, the hormonally active form of vitamin D_3_, has been being evaluated in clinical trials as an anticancer agent. However, currently available results are contradictory and not fundamentally deciphered. To the best of our knowledge, hypercalcemia caused by high‐dose calcitriol administration and its low bioavailability limit its anticancer investigations and translations. Here, we show that the one‐step self‐assembly of calcitriol and amphiphilic cholesterol‐based conjugates leads to the formation of a stable minimalist micellar nanosystem. When administered to mice, this nanosystem demonstrates high calcitriol doses in breast tumor cells, significant tumor growth inhibition and antimetastasis capability, as well as good biocompatibility. We further reveal that the underlying molecular antimetastatic mechanisms involve downregulation of proteins facilitating metastasis and upregulation of paxillin, the key protein of focal adhesion, in primary tumors.

**Abbreviations**
Cal: calcitriol
CFPE: carboxyfluorescein
CFPE‐mMNS@Cal: carboxyfluorescein (CFPE)‐labeled mMNS@Cal
Chol‐DNA: cholesterol‐DNA aptamer
Chol‐PEG_5K_
: cholesterol‐PEG_5K_
DDSs: drug delivery systems
ECM: extracellular matrix
mMNS: minimalist micellar nanosystem
mMNS@Cal: calcitriol containing minimalist micellar nanosystem
MMP‐2: matrix metallopeptidase‐2
MMP‐9: matrix metallopeptidase‐9
PDI: polydispersity index
TEM: transmission electron microscopy
VD_3_
: vitamin D_3_
VDR: vitamin D receptor

## 1 INTRODUCTION

Multiple preclinical studies have demonstrated that vitamin D receptor (VDR), a nuclear receptor that modulates transcription of target genes, exerts profound influences on the initiation and progression of human cancers.^1^, ^2^, ^3^, ^4^, ^5^ Vitamin D_3_ (VD_3_) from diets, supplements, or sun‐dependent synthesis in lower layers of skin epidermis, is not the biologically active ligand for VDR. It undergoes enzymatical metabolisms first in the liver and then in the kidney to become calcitriol (Cal), which binds to and then activates VDR.^6^ Breast cancer, as the most common cancer in women worldwide, in particular is highly related to VDR regulation.^7^, ^8^, ^9^

Multiple evidence from preclinical studies have clearly shown that (1) low plasma levels of Cal increase risks of breast cancer and metastasis,^7^, ^8^, ^9^ and (2) daily VD_3_ supplement is helpful for breast cancer prevention.^10^, ^11^, ^12^ The combinational treatments of Cal with cytotoxic chemotherapy, radiation, or other anticancer cytotoxic drugs showed additive or synergistic effects against breast cancer.^13^, ^14^, ^15^, ^16^, ^17^ For example, the combined treatment of Cal or its analogs with gefitinib significantly enhanced the antiproliferative activity of gefitinib in EGFR and HER2 positive breast tumors.^14^ On the other side, the question if Cal alone could be administered for breast cancer treatment, however, has not yet been systematically explored. Several studies showed promising but differing results.^18^, ^19^, ^20^ The IC_50_ of Cal was tested out to be around 10 nM on SUM‐229PE and MCF‐7 breast cancer cell lines during 6‐day in vitro culture in the study led by Martínez‐Reza et al.^20^ Haddur et al.^21^ found that IC_50_ of Cal on MCF‐7 and MDA‐MB‐231 cell lines after 24‐h incubation were around 50 μM. These in vitro studies conducted under different experimental conditions thus have not clearly and precisely revealed potentials of Cal on breast cancer treatment. An ongoing clinical phase 2 trail (ClinicalTrials.gov Identifier: NCT01293682) aiming to figure out effects of high‐dose Cal in breast cancer patients was started in 2010, clear conclusions however have not been made.

Excessive Cal administration can result in side effects including hypercalcemia, vascular calcification, and anaphylaxis, primarily because the drug shows nonselective distribution in vivo.^22^, ^23^, ^24^, ^25^, ^26^ One of the major challenges with Cal for breast cancer investigations therefore is achieving a sufficiently high drug concentration within cancer cells. Delivering Cal via nanoscale drug delivery systems (DDSs) may overcome this, as DDSs have often proven successful at targeting drugs to tumors via active targeting mechanisms or passive targeting mechanisms like the enhanced permeability and retention effect.^27^, ^28^ DDSs carrying vitamin D or its derivatives, including succinic acid‐based nanoparticles and liposomes, have recently been developed for cancer treatments, showing promising opportunities.^29^ In this study, we develop a micellar nanosystem to deliver Cal specifically into breast cancer cells, we then systematically evaluate potentials of Cal on breast cancer treatment.

## 2 RESULTS AND DISCUSSION

**FIGURE 1:**
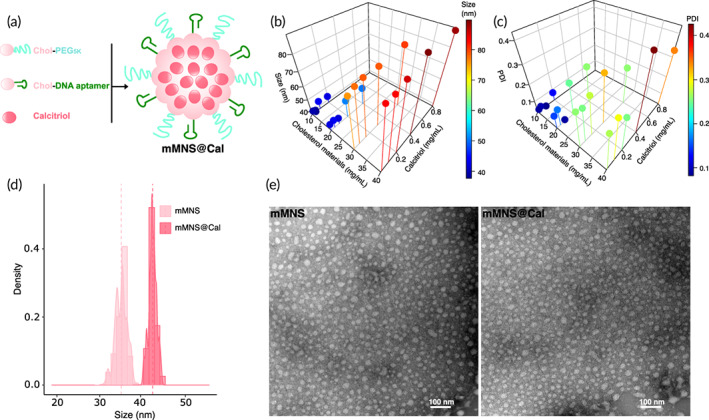
Characterization of micelles. (a) Schematic of the mMNS@Cal, which is formed via one‐step self‐assembly in an aqueous solution: the cholesterol parts of Chol‐PEG5K and Chol‐DNA form the hydrophobic core of the system while packing Cal molecules into it. (b) Size and (c) PDI of mMNS@Cal formed from different concentrations of cholesterol materials (the molar ratio of Chol‐PEG5K to Chol‐DNA is at (1) and Cal. Results are reported as mean values (*n* = 3). (d) Hydrodynamic size distributions and (e) TEM images of mMNS and mMNS@Cal. Scale bars = 100 nm

We synthesized a minimalist micellar nanosystem (mMNS) to deliver Cal specifically to breast cancer cells. mMNS self‐assembled from cholesterol‐PEG_5K_ (Chol‐PEG_5K_) and cholesterol‐DNA aptamer (Chol‐DNA) conjugates. Differing from drug delivery systems prepared from laboratory‐dominating special materials, both Chol‐PEG_5K_ and Chol‐DNA are widely and extensively used in research, and they are commercially available, which can thus facilitate the translation. Cal, as a fat‐soluble compound,^30^ can easily and stably be packed into the hydrophobic cholesterol core of mMNS (Figure 1a), forming the Cal containing mMNS—mMNS@Cal. The hydrophilic PEG_5K_ shell of mMNS@Cal can stabilize the system in vivo, prolonging its circulation in blood.^31^, ^32^ DNA aptamers targeting nucleolin on mMNS@Cal could direct the whole system into nucleolin‐overexpressing breast cancer cells.^33^, ^34^ This system thus allows us to investigate pharmacological effects of high intracellular Cal dose on breast cancer.

We controlled the molar ratio of Chol‐PEG_5K_ to Chol‐DNA at 1 to prepare mMNS. With varying the concentration of each component, particle size and polydispersity index (PDI) measured by dynamic light scattering are used as the criteria to screen out the good preparation. Along with increasing the concentration of cholesterol conjugates or Cal, sizes of mMNS@Cal increased (Figure 1b), while corresponding PDI of each preparation also increased (Figure 1c). This screening comparison leaded us to use 20 mg/ml cholesterol conjugates and 240.38 μM Cal as the final formula. The Cal encapsulation efficiency and loading efficiency with thus formula was measured to be 82.2% ± 2.6% and 32.8% ± 1.2%, respectively. mMNS@Cal prepared from this formula had its size at 42 ± 2.2 nm and PDI at 0.11 ± 0.03 nm, while the size and PDI of mMNS were 34 ± 5.1 and 0.09 ± 0.02 nm, respectively (Figure 1d). Both mMNS and mMNS@Cal had similarly negative zeta potentials at around −1.5 mV, which should be mainly contributed by DNA aptamers around the systems (Figure S1). Transmission electron microscopy (TEM) imaging further validated our preparations (Figure 1e). mMNS@Cal showed a good stability in the presence of serum at 37°C for 2 weeks, without big changes in size (Figure S2). Less than 10% of loaded Cal was released from mMNS@Cal during this 2‐week evaluation, further demonstrating its stability (Figure S3). This slow releasing profile of Cal from mMNS@Cal in vitro might reduce the release of Cal in blood circulation, mitigating side effects caused by nonselective distribution of Cal. After reaching tumor site and internalizing by tumor cells, the intracellular release profile of Cal would be accelerated by the lysosomes,^35^ leading to an immediate drug release. To confirm the containing of DNA aptamer around mMNS@Cal, we incubated mMNS@Cal with a Cy5‐modified complementary (to the sequence of DNA aptamer) DNA strand overnight under room temperature, which was then followed by washing unbound Cy5‐modified DNA strand away via dialysis. The result showed that mMNS@Cal had strong Cy5 signal (data not shown), indicating the existence of DNA aptamer on it.

**FIGURE 2:**
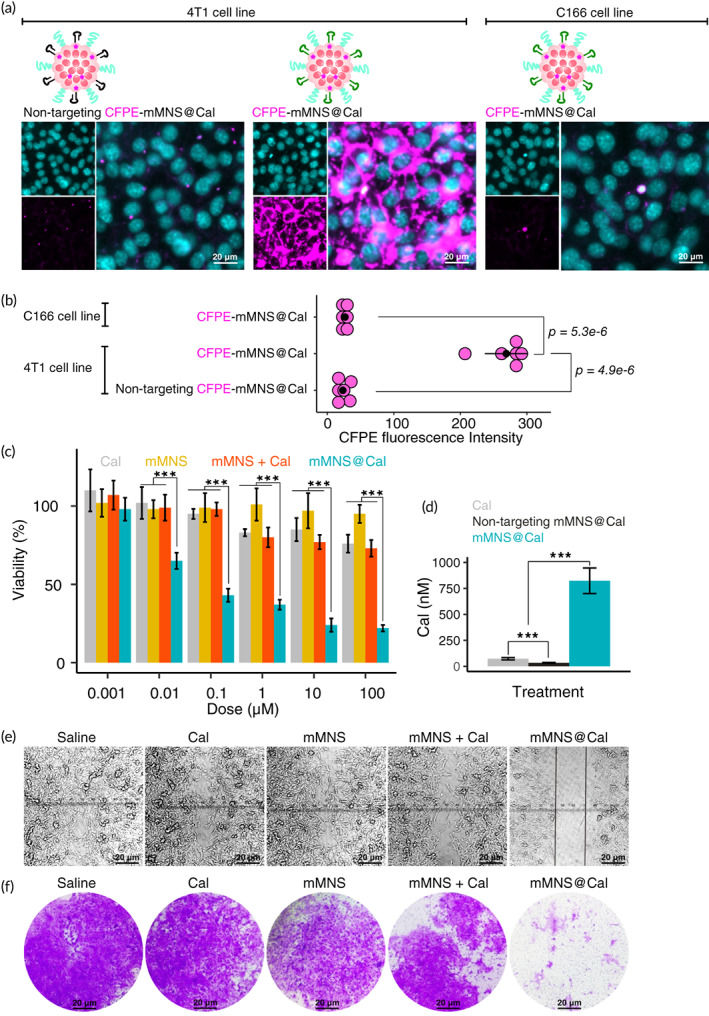
Cellular uptake, viability, and metastasis. (a) Uptake of CFPE‐labeled nontargeting mMNS@Cal by 4T1 cells, and uptake of CFPE‐labeled mMNS@Cal by 4T1 or C166 cells, imaged under confocal laser microscopy. Cell nucleus in cyan, CFPE in magenta. Scale bars = 20 μm. (b) Quantitative uptake measurements of CFPE‐labeled systems in 4T1 or C166 cells, measured by flow cytometry. Each point stands for the mean value of one biological replicate (*n* = 6). (c) Cell viability assay with indicated treatments on 4T1 cells. (d) Quantitative analysis of intracellular Cal after treatments for 4 h. Wound healing (e) and invasion (f) assays of 4T1 cells after indicated treatments. Scale bars = 20 μm. There are three biological replicates. *p < .05; ***p < .001

We firstly treated 4T1 breast cancer cells (with high nucleolin expression, which was confirmed by western blotting; Figure S4) with carboxyfluorescein (CFPE)‐labeled mMNS@Cal (CFPE‐mMNS@Cal) or nontargeting CFPE‐mMNS@Cal (which was prepared as same as CFPE‐mMNS@Cal but a random DNA sequence was used to replace the nucleolin‐targeting DNA aptamer) (Figure 2a). We observed that CFPE‐mMNS@Cal had a ~30 times higher intracellular delivery than nontargeting CFPE‐mMNS@Cal. Along with this, we compared the uptake efficacy of CFPE‐mMNS@Cal on 4T1 cells and C166 endothelial cells (as nucleolin negative cell line, which was confirmed by western blotting; Figure S4). It again showed a ~30 times higher CFPE fluorescent signal in 4T1 cells than it in C166 cells (Figure 2a,b). These observations indicated that DNA aptamers on mMNS@Cal can selectively mediate the nanosystem into cancer cells highly expressing nucleolin.

As the pharmacological result, IC_50_ of mMNS@Cal (0.04 ± 0.01 μM) was much lower than IC_50_ of Cal (>100 μM) or IC_50_ of the mixture of mMNS and Cal (Figure 2c). The empty system mMNS itself did not show cytotoxicity. Our quantitative measurement of intracellular Cal on 4T1 cells treated with 1‐μM Cal showed that, compared with free Cal, mMNS@Cal significantly increased intracellular Cal by around 28 folds (Figure 2d). These results further proved that nucleolin‐targeting DNA aptamer locating surround the mMNS@Cal promote the intracellular delivery of Cal.

Wound healing assay and transwell‐based invasion assay are usually conducted to evaluate metastasis of cancer cells in vitro.^36^ We thus carried out these two assays (Figure 2e,f) to study if high intracellular dose of Cal can impact the metastatic properties of breast cancer cells. At the concentration of 1 μM, Cal itself showed no effect on inhibiting the wound healing and cancer cell invasion. Apart from this, neither could the delivery system mMNS itself nor the mixture of mMNS and Cal significantly slow down the wound healing and transwell‐based invasion. Nonetheless, mMNS@Cal showed great metastasis‐inhibiting potentials on these two assay models. These in vitro experimental results together indicate that, at low concentration of 1 μM, Cal does have antimetastatic pharmacological effects, which however is restrained by its poor uptake by cells.

**FIGURE 3:**
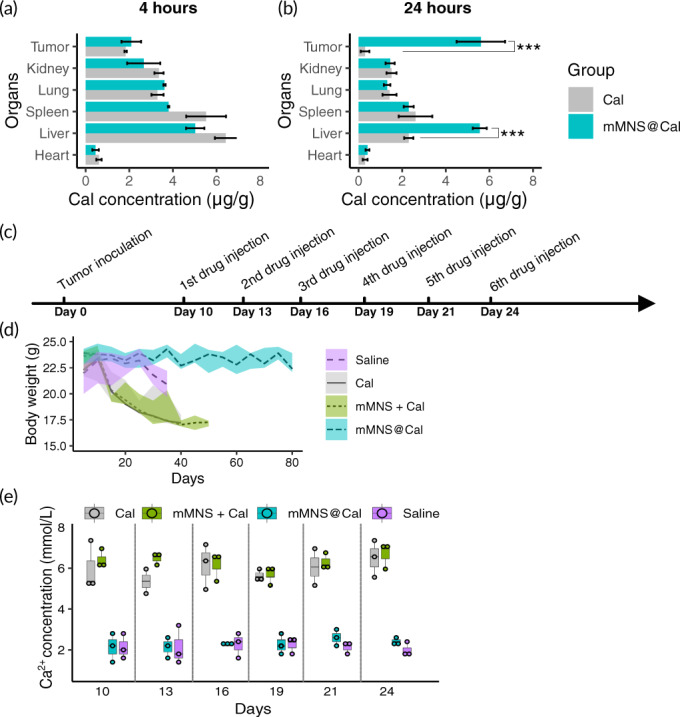
Cal biodistribution and biosafety. Cal amount in organs and tumors at 4 h (a) and 24 h (b) after intravenous administration with Cal or mMNS@Cal to tumor‐bearing mice. (c) Tumor inoculation and treatment schedule for biosafety evaluation. (d) Body weight records of tumor‐bearing mice receiving different treatments. (e) Blood calcium measurements along days of tumor‐bearing mice receiving different treatments. ***p < .001

To assess if mMNS@Cal could specifically deliver high‐dose Cal to tumor cells in vivo, we intravenously administrated it, at 5 mg·kg^−1^ Cal, to mice orthotopically bearing 4T1 tumor. We then sacrificed mice at specific time points and quantified Cal in tumors and organs using high performance liquid chromatography (HPLC) measurement. For Cal itself, at 4 h, it clearly showed that liver was the main biodistributing organ of Cal from both free Cal and mMNS@Cal administrations (Figure 3a). There was no significant difference of tumorous Cal, which was around 2 μg·g^−1^, between free Cal and mMNS@Cal administrations. At 24th hour, tumors from mice treated with mMNS@Cal had Cal content at 5.61 ± 0.11 μg·g^−1^, whereas only 0.3 ± 0.11 μg·g^−1^ Cal was tested out in tumors from mice treated with free Cal (Figure 3b). This around 18‐fold increase of Cal in tumors indicated that mMNS@Cal can deliver high‐dose Cal to tumors in vivo. Compared with free Cal administration, around 2.4 folds more Cal stayed in livers of mMNS@Cal treating mice at the 24th hour.

Cal, as the metabolite of VD_3_, is thought to be beneficial for health on many aspects. However, taking overdoses of Cal can lead to hypercalcemia by increasing the absorption of Ca^2+^ in the kidneys, causing damages to bones, kidneys, heart, and brain.^37^ We then evaluated the associated side effects of multiple times of Cal injection (5 mg·kg^−1^ bw) on 4T1 tumor‐bearing mice (Figure 3c). Compared to mice injected with Cal or the mixture of mMNS and Cal, the safety of mMNS@Cal treatment was highlighted by alleviated reductions of mice body weights (Figure 3d). More importantly, we measured blood Ca^2+^ levels at different time points to assess risks of hypercalcemia. It showed that multiple times of Cal injection caused much higher blood Ca^2+^ levels than the hypercalcemia threshold level (2.6 mmol·L^−1^).^38^ Nonetheless, under the same Cal dosages, mice injected with mMNS@Cal kept their blood Ca^2+^ levels within safe ranges (Figure 3e).

**FIGURE 4:**
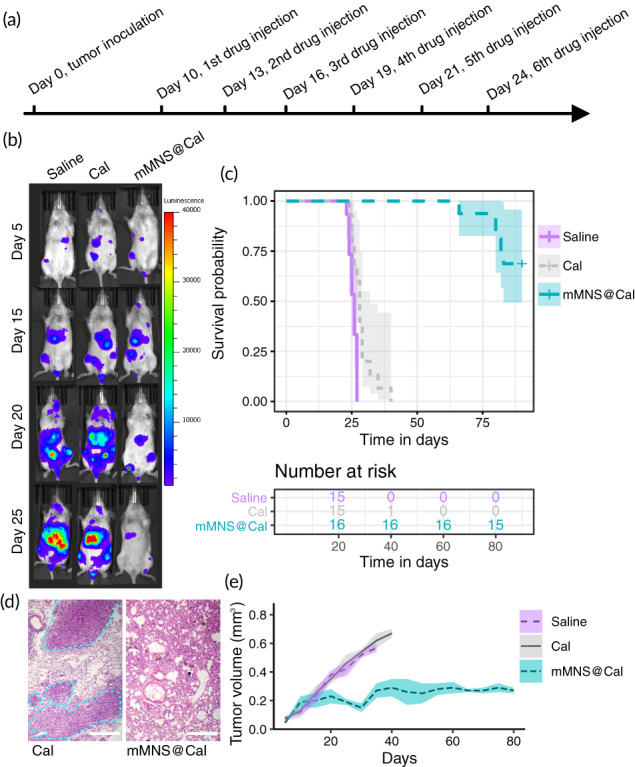
In vivo antimetastasis and antitumor evaluation. (a) Tumor inoculation and treatment schedule. (b) Bioluminescence imaging to track the metastasis on mice treated with saline, Cal or mMNS@Cal. (c) Kaplan–Meier tumor‐inoculated mouse survival curves. (d) Representative hematoxylin and eosin stain sections of lung tissues from mice treated with Cal or mMNS@Cal. Tumor areas are highlighted within dotted lines. (e) Tumor volume growth curves. Scale bar = 100 μm

To evaluate the translation of mMNS@Cal for in vivo treatments, luciferase‐expressing 4T1 cell line was used to establish tumor model in mice and metastasis was thus monitored via bioluminescence imaging. During the treatment (Figure 4a), mMNS@Cal showed significant antimetastatic effects (Figure 4b), prolonging the median survival of mice by more than 50 days (vs. Cal group) (Figure 4c). Mice treated with Cal gradually suffered severe lung metastasis (Figure 4d) and died. Besides its antimetastatic capacity, mMNS@Cal treatment also effectively inhibited the growth of primary tumors (Figure 4e). These in vivo experimental results together confirmed that Cal has potentials for metastatic breast cancer treatment which however needs a high enough intratumor dose.

**FIGURE 5:**
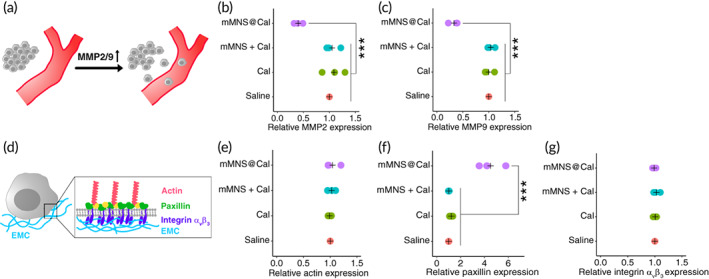
In vivo metallopeptidases and focal adhesion‐relative proteins detection. (a) The upregulation of MMP2 or MMP facilitate metastasis of breast cancer. (b) MMP2 and (c) MMP9 detections in tumors. (d) Main components illustration of focal adhesion. (e) Actin, (f) paxillin, and (g) integrin αvβ3 expression detections in tumors treated as indicated. ***p < .001

We explored the antimetastatic mechanisms of mMNS@Cal. The detachment of cancer cells from the primary tumor is the first stage of metastasis. During this stage, cancer cells have to overcome several restrictions, including tumorous structural obstacles, integrin‐mediated cell matrix adhesions, and other factors within tumor extracellular matrix (ECM).^39^, ^40^, ^41^, ^42^ Results above drove us to investigate if the observed antimetastatic effects of mMNS@Cal are related to this. We thus first detected matrix metallopeptidase‐2 (MMP‐2) and matrix metallopeptidase‐9 (MMP‐9) in extracts from differently treated tumors. Tumor cells can actively express both MMP‐2 and MMP‐9, which then might facilitate degrading and remodeling ECM during the early stage of breast cancer metastasis (Figure 5a).^43^, ^44^, ^45^ It could be inferred from enzyme‐linked immunosorbent assays that mMNS@Cal treatment significantly inhibited the expressions of MMP‐2 (Figure 5b) and MMP‐9 (Figure 5c), whereas neither did Cal treatment nor mMNS plus Cal treatment cause obvious changes as compared to tumors treated with saline. We also quantified tumorous expressions of integrin α_v_β_3_, actin and paxillin, which together are main scaffolding components of focal adhesion between cells and ECM.^46^ The structure of focal adhesion (Figure 5d) has been revealed to limit the detachment of tumor cells from primary tumors during the early stage of metastasis.^47^, ^48^, ^49^ Our results showed that, although our dosage regimens did not result in fluctuations of integrin α_v_β_3_ (Figure 5e) and actin (Figure 5g), tumors treated by mMNS@Cal had much more paxillin than tumors treated by Cal or the mixture of mMNS and Cal (Figure 5f). Specifically, mMNS@Cal improved the paxillin amount by 4.33 ± 0.88 folds (vs. Cal) and 4.81 ± 0.73 folds (vs. mMNS plus Cal), respectively. These protein profiles reveal that via downregulating MMP2/9, maintaining focal adhesion and other potential molecular mechanisms, mMNS@Cal restricted cancer cells within primary tumor, reducing metastasis.

## 3 CONCLUSIONS

Thus, with all these results, it showed that our one‐step self‐assembled mMNS@Cal, with a high loading efficiency, can pack the hormonally active form of vitamin D into its cholesterol core. Directed by DNA aptamers modified around it, mMNS@Cal selectively delivered Cal into nucleolin‐expressing breast cancer cells, both in vitro and in vivo, with high intracellular doses. We then proved that high intracellular doses of Cal reduced the growth of primary breast cancer and weakened metastasis. Our mechanism studies showed us that proteins facilitating tumor metastasis, including MMP‐2 and MMP‐9, were downregulated by mMNS@Cal. Besides, paxillin, as the key component of the focal adhesion complex, was upregulated by mMNS@Cal. These together implied that the molecular antimetastasis mechanism of mMNS@Cal is at least related to its ability to limit cancer cells' detachment from the primary tumor. More importantly, to fully unlock the anticancer and antimetastasis pharmacological functions of Cal, a reliable intracellular nanoscale drug delivery system like the system we used in this work will be needed.

## 4 MATERIALS AND METHODS

### 4.1 Blank micelles preparation

We used the thin‐film hydration method as described in previous study to prepare micelles.^50^ Two hundred milligrams of Chol‐PEG_5K_ (Integrated DNA Technologies) and Chol‐Apt (Integrated DNA Technologies) (molar ratio of Cho‐PEG5k to Cho‐Apt was 1:1) were dissolved in 10 ml of absolute ethanol and 1 ml of chloroform in a round‐bottomed flask. The nucleolin‐targeting DNA sequence is: GGTGGTGGTGGTTGTGGTGGTGGTGG. The solvents were removed using rotary evaporation to obtain a thin film. The system was then dried overnight to remove any residual solvent. The resultant thin film was hydrated with 10 ml of water (preheated at 55°C) and mixed thoroughly on a 55°C water bath for 5 min. This collected solution was then bath sonicated for 10 min and left to settle at room temperature for a further 10 min. The solution was then passed through a sterile 0.22‐μm filter to remove any large aggregates.

### 4.2 Cal‐loaded micelles

The workflow was same as the preparation of blank micelles except for the addition of Cal (amounts were indicated in Figure 1b,c) (D1530; Sigma‐Aldrich) to the absolute ethanol and chloroform solution. The optimized ratio between blank micelle and Cal was selected with regards to size, PDI, encapsulation efficacy, and release profile of Cal. The encapsulation efficacy of Cal was measured according to Nicolas et al.^30^ and the formula is shown as

$$\mathrm{Cal}\;\text{encapsulation efficiency}=\left(\text{total}\;\mathrm{Cal}\;\text{added}-\text{free non}-\text{encapsulted}\;\mathrm{Cal}\right)\;÷\left(\text{total}\;\mathrm{Cal}\;\text{added}\right)\times 100.$$

#### 4.2.1 Cal release study

Release of Cal from mMNS@Cal was carried out by a centrifugation method^30^ at 37°C for 2 weeks. Phosphate buffered saline (PBS) (pH 7.4) was used as the release medium. mMNS@Cal suspension was diluted in 50 ml of release buffer to a final Cal concentration at 9.62 μM and placed under moderate magnetic stirring (100 rpm) at 37°C. At specific time intervals, samples of 1 ml were withdrawn, centrifuged for 30 min, and the drug released was quantified in the supernatant by HPLC.

### 4.3 Characterizations of the preparations

Size distributions were measured using a laser particle size analyzer (Malvern Nano ZS) with preparation suspension diluted in Milli‐Q water at room temperature. Zeta potentials were investigated using a laser particle size analyzer (Malvern Nano ZS). Results were expressed as the average of three measurements. The size distribution is given by polydispersity index (PDI).

### 4.4 Transmission electron microscopy

A 5‐μl aliquot of samples was spotted on a glow discharged, carbon‐coated, formvar resin grid (Electron Microscopy Sciences) for 30 s before blotting on a filter paper, and then stained with aqueous uranyl formate solution (2% [wt/vol]) for 30 s. Then, the uranyl formate solution was removed by filter paper blotting. The stained sample was imaged using an FEI Morgagni 268 transmission electron microscope at 80 kV with magnifications 12 K.

### 4.5 Cell culture

Murine breast cancer 4T1 cells, luciferase‐expressing 4T1 cells, and murine endothelial C166 cells were purchased from ATCC and cultured in RPMI 1640 Medium (Sigma‐Aldrich). All cultures were supplemented with 10% fetal bovine serum (Gibco), 100 U/ml streptomycin, and 100 U/ml penicillin (Gibco) in a humidified atmosphere of 5% CO_2_ at 37°C.

### 4.6 Western blotting

4T1 cells and C166 cells cultured in 60‐mm dishes (5 × 10^6^ cells per dish) were placed on ice and washed with ice‐cold PBS. 0.5 ml of ice‐cold lysis buffer containing protease inhibitors (Sigma‐Aldrich, MCL1) was added to the dish to homogenized and lyses cells. Protein concentrations were determined by Pierce™ Coomassie (Bradford) Protein Assay Kit (Thermo Fisher Scientific, 23200). 20 μg of proteins was mixed in 1:1 ration with 2X Laemmli buffer, denatured by boiling at 100°C for 5 min, and then loaded and run on 10% sodium dodecyl sulfate–polyacrylamide gel electrophoresis. The gels were transferred to polyvinylidene difluoride membranes, and then incubated with nucleolin monoclonal antibody (Thermo Fisher Scientific, ZN004) at 4°C overnight. The next day membranes were washed with Pierce™ 20X TBS Tween™ 20 Buffer (Thermo Fisher Scientific, 28360) and incubated for 1 h at room temperature with horseradish peroxidase (HRP)‐conjugated secondary antibody (Thermo Fisher Scientific, 62–6520). The binding was detected by Immobilon Western HRP Substrate (Millipore, WBKLS0100) using the ChemiDoc MP system (Bio‐Rad Laboratories).

### 4.7 Cellular uptake of CFPE‐labeled micelles

4T1 cells or C166 cells were seeded into six‐well plates (1 × 10^5^ cells per well) and cultured overnight. Then, CFPE‐labeled micelles were added to cells, at the final CFPE concentration of 2 mg/ml, for another 4‐h incubation. For qualitative analysis, cells were fixed with 4% (vol/vol) paraformaldehyde. Nucleus were stained with 4′,6‐diamidino‐2‐phenylindole. Fluorescence imaging was performed using confocal microscopy (TCS SP5 AOBS confocal microscopy system; Leica). For quantitative analysis, cells were digested by 0.25% Trypsin–EDTA (Gibco), washed with clod 1× PBS and collected by centrifugation at 1500*g* for 5 min. Cells were finally resuspended in 500‐μl 1× PBS. CFPE fluorescence of cells was measured using flow cytometry (Cytomics™ FC 500, Beckman Coulter).

### 4.8 Quantitative Cal measurement in cells

Cells cultured in six‐well plates (1 × 10^5^ cells per well) were incubated with preparations (at 1‐μM Cal) for 4 h. Cells were then washed by cold 1× PBS and lysed by Cell Lysis Buffer (Invitrogen). Methanol was used to extract Cal from the whole cell lysates. After shaking and centrifuging, the supernatant fractions were collected and Cal in it was analyzed by HPLC according to previous publications.^51^

### 4.9 Cell viability

We took ATP‐based luminescent cell viability assay with the kit named CellTiter‐Glo Luminescent Cell Viability Assays (Promega). In brief, 5 × 10^4^ cells per well were seeded in a 96‐well opaque white polystyrene microplate (Corning) and cultured overnight. Cells were treated with various concentrations, in the term of calcitriol (from 0.001 to 100 μM), of preparations. After 48‐h incubation, plates were equilibrated at room temperature for half hour. 100 μl of CellTiter‐Glo reagent (Promega) was added to each well, followed by 2‐min incubation on an orbital shaker to lyse cells. Plates were then incubated at room temperature for 10 min to stabilize the luminescent signal. Finally, the luminescence was quantified on a multimode microplate reader (Thermo Fisher Scientific Varioskan Flash). Wells containing medium only were used as the background luminescence

$$\%\text{viable cell}=\left(\text{luminescence of sample}-\text{luminescence of background}\right)\;÷\left(\text{luminescence of}\;\mathrm{PBS}-\text{luminescence of background}\right)\times 100.$$

#### 4.9.1 In vitro antimetastatic assay

For wound healing assay, 1 × 10^6^ 4T1 cells were cultured to confluence in six‐well plates. After scratching a straight line through the cell layer with a sterile 200‐μl pipette tip, cells were treated with different preparations (with the concentration of Cal at 1 μM) for 24 h. Images of wound closure were then captured.

For transwell invasion assay, 1 × 10^6^ cells grew in the top chamber with 50‐μl Matrigel‐coated membranes (pore size at 8 μm). After treating with different preparations (with the concentration of Cal at 1 μM) for 12 h, cells that did not invade through the Matrigel‐coated membranes were removed. Invasive cells migrating to the lower surface of the membranes were fixed with 4% paraformaldehyde and then stained with crystal violet for 20 min.

### 4.10 Breast tumor model

Female BALB/c mice were purchased from Experimental Animal Center of Sichuan University (China). All animal studies were performed under the experimental guidelines of the Animal Experimentation Ethics Committee of Sichuan University. For the establishment of breast tumor models, 2 × 10^6^ luciferase‐expressing 4T1 cells were orthotopically implanted into the mammary fat pad of mice. Treatment started at the 10th day after tumor inoculation, and the tumor sizes at the 10th day were around 0.1 mm^3^. Tumor growth and metastasis were monitored by measuring sizes of primary tumor and bioluminescence imaging. All animal experiments were approved by the Committee on the Ethics of Animal Experiments of the Sichuan University.

### 4.11 Antimetastatic and antitumor assay

After tumor cell inoculation, we started treatments on the 10th day. We randomly divided mice bearing luciferase‐expressing 4T1 tumors into different experimental groups (10 mice per group). Preparations (Cal at 10 mg·kg^−1^) were administrated via tail vein injection (every 3 days for six times). To monitor tumor metastasis, mice were injected with d‐luciferin Firefly potassium salt (Caliper Life Sciences) before bioluminescence imaging under an in vivo Spectrum system (Caliper) with 60‐s exposure time. Tumor volume, body weight, and lifetime were also recorded. Volume of tumor was calculated according to formula: volume (mm^3^) = 1/2 L (length) × B (width).^2^

### 4.12 Protein assay

We quantitatively measured interested proteins by specialized Elisa kits. In vitro, after treatment with 1‐μM Cal, cells were harvested and lysed in cell lysis buffer containing protease inhibitors. Then, the whole cell lysis was detected by ELISA Kits (Mouse MMP‐2 Elisa Kit (RAB0366; Sigma‐Aldrich); Mouse pro‐MMP‐9 Elisa Kit (RAB0373, Sigma‐Aldrich); ACTB/Beta Actin Elisa Kit (LS‐F10737‐1, LSBio); PXN/Paxillin Elisa Kit (LS‐F21248‐1, LSBio); Mouse Integrin Alpha V Beta 3 (ITGAVB3) Elisa Kit (MBS9362139_48wells, MyBioSource)) according to corresponding instructions. Ex vivo, three mice/group were sacrificed on the 20th day during the treatment and their tumors were collected. The tumors were diced into small pieces, and then lysed in cell lysis buffer containing protease inhibitors. After this, proteins in the tumor tissue lysis were analyzed by Elisa kits.^52^

### 4.13 Blood calcium level measurement

During our treatment protocol, blood samples of mice (three mice per group) were collected from tail at specified days. Serum was then separated via centrifugation for calcium measurement. A fluorescence‐based Calcium Assay Kit (ab112129; Abcam) was used, according to the product instruction, to read calcium levels in serum.^52^

### 4.14 Cal biodistribution

During our treatment protocol, after 24 h of the 6th drug injection, three mice per group were sacrificed and perfused (via heart) with cold PBS to collect organs. Organs were then homogenized within methanol. After centrifugation, supernatant was collected for Cal quantification by HPLC methods published before.^54^

### 4.15 Statistics

All the statistical analysis were carried out in R. When not otherwise stated, results were shown as mean values ± *SD*. Data were analyzed using two‐tailed Student's *t* tests between two groups and one way analysis of variance followed by Turkey posttests among multiple groups. *p* value less than .05 were considered significant.

## AUTHOR CONTRIBUTIONS

**Jiaye Liu:** Conceptualization (lead); data curation (lead); formal analysis (lead); investigation (lead); methodology (lead); project administration (lead); software (lead); validation (lead); visualization (lead); writing – original draft (lead); writing – review and editing (lead). **Junyi Shen:** Data curation (lead); formal analysis (lead); funding acquisition (lead); investigation (equal); methodology (lead); project administration (lead); software (equal). **Chunyang Mu:** Conceptualization (equal); formal analysis (equal); investigation (lead); methodology (lead); project administration (lead); software (equal). **Yang Liu:** Conceptualization (equal); formal analysis (equal); investigation (lead); methodology (lead); project administration (lead). **Dongsheng He:** Formal analysis (equal); funding acquisition (lead); investigation (lead); resources (equal); validation (lead); visualization (lead). **Han Luo:** Formal analysis (lead); funding acquisition (lead); investigation (equal); validation (equal); visualization (equal). **Wenshuang Wu:** Formal analysis (lead); funding acquisition (lead); investigation (equal); methodology (lead); resources (lead); validation (equal). **Xun Zheng:** Formal analysis (lead); investigation (lead); methodology (equal); resources (equal); validation (lead). **Yi Liu:** Formal analysis (lead); funding acquisition (lead); investigation (lead); methodology (equal); resources (lead); validation (equal). **Sunrui Chen:** Formal analysis (lead); investigation (lead); methodology (equal); resources (equal); validation (equal). **Qiuwei Pan:** Formal analysis (equal); investigation (lead); methodology (equal); validation (equal); writing – original draft (lead). **Yiguo Hu:** Formal analysis (equal); investigation (equal); methodology (lead); validation (equal); writing – original draft (lead); writing – review and editing (lead). **Yinyun Ni:** Formal analysis (equal); investigation (equal); methodology (lead); validation (lead). **Yang Wang:** Conceptualization (equal); data curation (equal); formal analysis (equal); methodology (lead). **Yong Liu:** Formal analysis (lead); investigation (equal); methodology (equal); supervision (lead); validation (lead). **Zhihui Li:** Formal analysis (lead); funding acquisition (lead); investigation (lead); methodology (lead); resources (lead); supervision (lead); validation (lead); writing – original draft (lead); writing – review and editing (lead).

## CONFLICT OF INTERESTS

The authors declare that there are no conflict of interests.

### PEER REVIEW

The peer review history for this article is available at https://publons.com/publon/10.1002/btm2.10263.

## Supporting information


**Appendix** **S1**: Supporting Information

## Data Availability

The raw/processed data required to reproduce these findings cannot be shared at this time due to technical or time limitations.
