# Supplementary material for: High‐dose vitamin D metabolite delivery inhibits breast cancer metastasis
Source: Bioeng Transl Med. 2021 Oct 27;7(1):e10263. doi: 10.1002/btm2.10263 (PMC8780911; doi:10.1002/btm2.10263)
Supplement: Supplementary file 1 — Appendix S1: Supporting Information [file BTM2-7-e10263-s001.docx]

*Supporting Information*

**High Dose Vitamin D Metabolite Delivery Inhibits Breast Cancer Metastasis**

Jiaye Liu^1,2,8,9^, Junyi Shen^3^, Chunyang Mu^3^, Yang Liu^1,2^, Dongsheng He^4^, Han Luo^1,2^, Wenshuang Wu^1,2^, Xun Zheng^1,2^, Yi Liu^5^, Sunrui Chen^6^, Qiuwei Pan^7^, Yiguo Hu^8^, Yinyun Ni^9^, Yang Wang^10^, Yong Liu^11#^, Zhihui Li^1,2#^

^1^Department of Thyroid and Parathyroid Surgery, West China Hospital, Sichuan University, Chengdu, Sichuan, China.

^2^Laboratory of Thyroid and Parathyroid diseases, Frontiers Science Center for Disease-Related Molecular Network, West China Hospital, Sichuan University, Chengdu, Sichuan, China.

^3^Department of Liver Surgery & Liver Transplantation Center, West China Hospital, Sichuan University, Chengdu, Sichuan, China.

^4^Department of Pharmaceutics, School of Pharmacy, China Pharmaceutical University, Nanjing, Jiangsu, China.

^5^Department of Rheumatology and Immunology, Rare Disease Center, West China Hospital, Sichuan University, Chengdu, Sichuan, China.

^6^Shanghai OneTar Biomedicine, Shanghai, China.

^7^Department of Gastroenterology and Hepatology, Erasmus MC-University Medical Center, Rotterdam, The Netherlands.

^8^State Key Laboratory of Biotherapy and Cancer Center, West China Hospital, Sichuan University and Collaborative Innovation Center, Chengdu, Sichuan, China.

^9^Department of Respiratory and Critical Care Medicine, Center of Precision Medicine, Precision Medicine Key Laboratory of Sichuan Province.

^10^Department of Medical Biochemistry and Biophysics, Karolinska Institute, Stockholm, Sweden.

^11^Department of Gastroenterological Surgery, West China Hospital, Sichuan University, Chengdu, China

***To whom correspondence should be address:**

Yong Liu, Prof. MD. PhD, E-mail: beckmanliuyong@163.com; Department of Gastroenterological Surgery, West China Hospital, Sichuan University, Chengdu 610041, Sichuan, China

Zhihui Li, Prof. MD. PhD, Email: [rockoliver@vip.sina.com](mailto:rockoliver@vip.sina.com), Department of Thyroid and Parathyroid Surgery, West China Hospital, Sichuan University, Chengdu 610041, Sichuan, China.

**Fig. S1.** Zeta potential distributions of mMNS and mMNS@Cal.

**Fig. S2.** The fluctuation in particle sizes of mMNS and mMNS@Cal in 50% FBS.

**Fig. S3.** Cal release from mMNS@Cal at different PBS with pH 7.4 over 2 weeks.


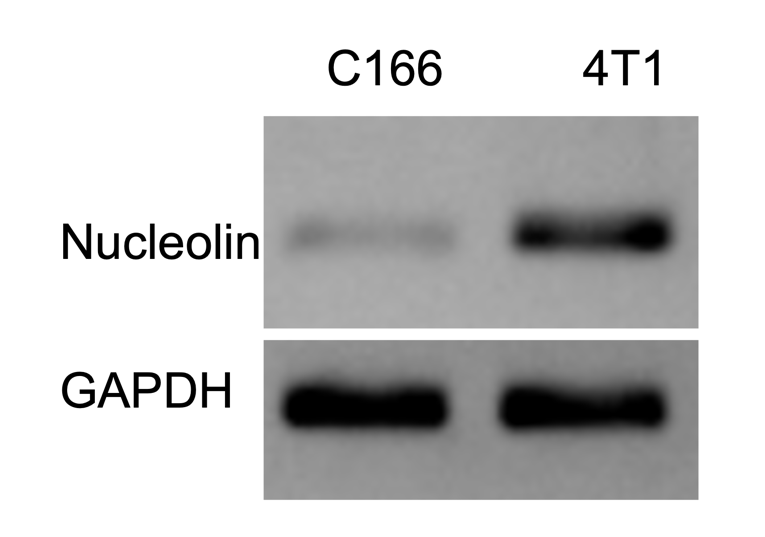


**Fig. S4.** Nucleolin expression in C166 cell line and 4T1 cell line.
